# Transcriptional profiling of *Pseudomonas aeruginosa* and *Staphylococcus aureus* during in vitro co-culture

**DOI:** 10.1186/s12864-018-5398-y

**Published:** 2019-01-10

**Authors:** Mikaël Tognon, Thilo Köhler, Alexandre Luscher, Christian van Delden

**Affiliations:** 1 Transplant Infectious Diseases Unit, University Hospitals of Geneva, Geneva, Switzerland; 2 Department of Microbiology and Molecular Medicine, University of Geneva, 1, rue Michel Servet, CH-1211 Genève 4, Switzerland

**Keywords:** *Pseudomonas aeruginosa*, *Staphylococcus aureus*, Transcriptome, RNAseq, Bacterial competition

## Abstract

**Background:**

Co-colonization by *Pseudomonas aeruginosa* and *Staphylococcus aureus* is frequent in cystic fibrosis patients. Polymicrobial infections involve both detrimental and beneficial interactions between different bacterial species. Such interactions potentially indirectly impact the human host through virulence, antibiosis and immunomodulation.

**Results:**

Here we explored the responses triggered by the encounter of these two pathogens to identify early processes that are important for survival when facing a potential competitor. Transcriptional profiles of both bacteria were obtained after 3 h co-culture and compared to the respective mono-culture using RNAseq. Global responses in both bacteria included competition for nitrogen sources, amino acids and increased tRNA levels. Both organisms also induced lysogenic mechanisms related to prophage induction (*S. aureus*) and R- and F- pyocin synthesis (*P. aeruginosa*), possibly as a response to stress resulting from nutrient limitation or cell damage. Specific responses in *S. aureus* included increased expression of de novo and salvation pathways for purine and pyrimidine synthesis, a switch to glucose fermentation, and decreased expression of major virulence factors and global regulators.

**Conclusions:**

Taken together, transcriptomic data indicate that early responses between *P. aeruginosa* and *S. aureus* involve competition for resources and metabolic adaptations, rather than the expression of bacteria- or host-directed virulence factors.

**Electronic supplementary material:**

The online version of this article (10.1186/s12864-018-5398-y) contains supplementary material, which is available to authorized users.

## Background

Since the recent advent of culture-independent analyses of complete microbial populations, microbe-host-microbiota interactions have opened new avenues for the study of infectious diseases. In this respect, detrimental and beneficial interactions between co-colonizing bacteria may influence the development and persistence of co-infections. Co-infection by different pathogenic bacterial species often increases the severity of the underlying condition, and are more difficult to treat [1]. For instance, *Pseudomonas aeruginosa* and *Staphylococcus aureus* are frequently co-isolated in leg ulcers [2], rhino-sinusitis [3], burn wounds and cystic fibrosis (CF) [4, 5]. Studies on animal models have shown that co-infection with these two pathogens leads to delayed wound repair and increased antibiotic resistance in mice [6], and increased mortality in flies [7].

While *S. aureus* is the dominant bacterial species in the lungs of young CF-patients, *P. aeruginosa* is dominant during adulthood [8]. Co-infection by *P. aeruginosa* and *S. aureus* frequently occurs and is associated with poor patient prognosis [5, 9]. However, data from in vitro and in vivo studies on the interaction between these pathogens remain scarce. Some reports show evidence for increased virulence of co-colonizing strains and decreased antibiotic treatment efficiencies [10, 11], while other studies describe commensal interactions between *P. aeruginosa* and *S. aureus* [12, 13]. These studies compared well-characterized laboratory strains of *P. aeruginosa* to clinical isolates of *P. aeruginosa* that have evolved within the CF-lung where they might interact with the co-colonizing *S. aureus*. They showed that laboratory strains of *P. aeruginosa* such as PA14 and early isolates from CF-patients outcompete *S. aureus*, while isolates after years of colonization have lost their ability to outcompete *S. aureus* in co-cultures [13]. This suggests that host-microbe or microbe-microbe adaptations occur during chronic colonization, which may subsequently influence infection progression and outcome.

Microbes can engage in mutualistic, neutral or antagonistic interactions, when sharing the same environment. During the infection, bacteria encounter nutrient poor environments leading to competition for resources and to antibiosis, although cooperative behaviors have also been described [14]. Carbon and nitrogen sources as well as iron are essential nutrients, which are often limited in the host. Thus nutrient scavenging is a widely used strategy among bacteria [15–17]. Antibiosis, which is defined by detrimental interactions between at least two species, usually involves the synthesis of antimicrobial molecules that inhibit growth or kill other bacterial species [18]. Finally, cooperation between bacterial species also occurs, but rather governs the interactions within the microbiota [19]. Interactions between *P. aeruginosa* and *S. aureus* have been reported from both in vitro and in vivo conditions. One prominent example is the inhibition of the cytochrome b mediated e-transport, mediated by 2-heptyl-4-quinolone *N*-oxide (HQNO) produced by *P. aeruginosa* [20], resulting in formation of small colony variants by *S. aureus* [21]. Furthermore, *P. aeruginosa* can lyse *S. aureus* through the action of the secreted LasA protease [22], allowing *P. aeruginosa* to get access to iron [23, 24]. Studies have also reported cooperative behaviors, in which *P. aeruginosa* protects *S. aureus* from phagocytosis by *Dictyostelium discoideum* through formation of mixed biofilms [25], and commensal interactions with adapted *P. aeruginosa* CF-isolates [12, 13, 26].

In this study, we present data from dual transcriptome experiments, which examine the early responses occurring after 3 h co-incubation in both *P. aeruginosa* and *S. aureus*. Our data reveal metabolic changes mainly in nucleotide and nitrogen metabolism, decreased expression of virulence factors and induction of phage-related genes in both bacteria. Our results are in agreement with an initial competition for nutrient resources rather than a direct interspecies competition response.

## Results

### Transcriptional responses in *P. aeruginosa* and *S. aureus*

**Fig. 1:**
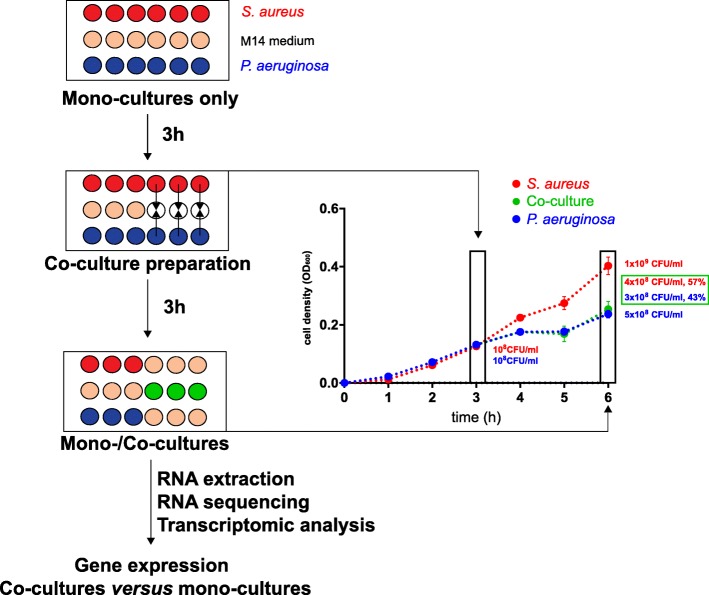
Experimental setup for culture preparation and RNA extraction. *S. aureus* and *P. aeruginosa* strains were first grown separately in microtiter plates under static conditions and then mixed at the same proportion to prepare the co-cultures. Plate counts show no significant differences between replicates. Proportion of *P. aeruginosa* and *S. aureus* in co-cultures. Shown are average values of thre three replicates with standard deviations at the beginning of the co-culture (*t* = 3 h) and after 3 h of incubation (*t* = 6 h)

**Fig. 2:**
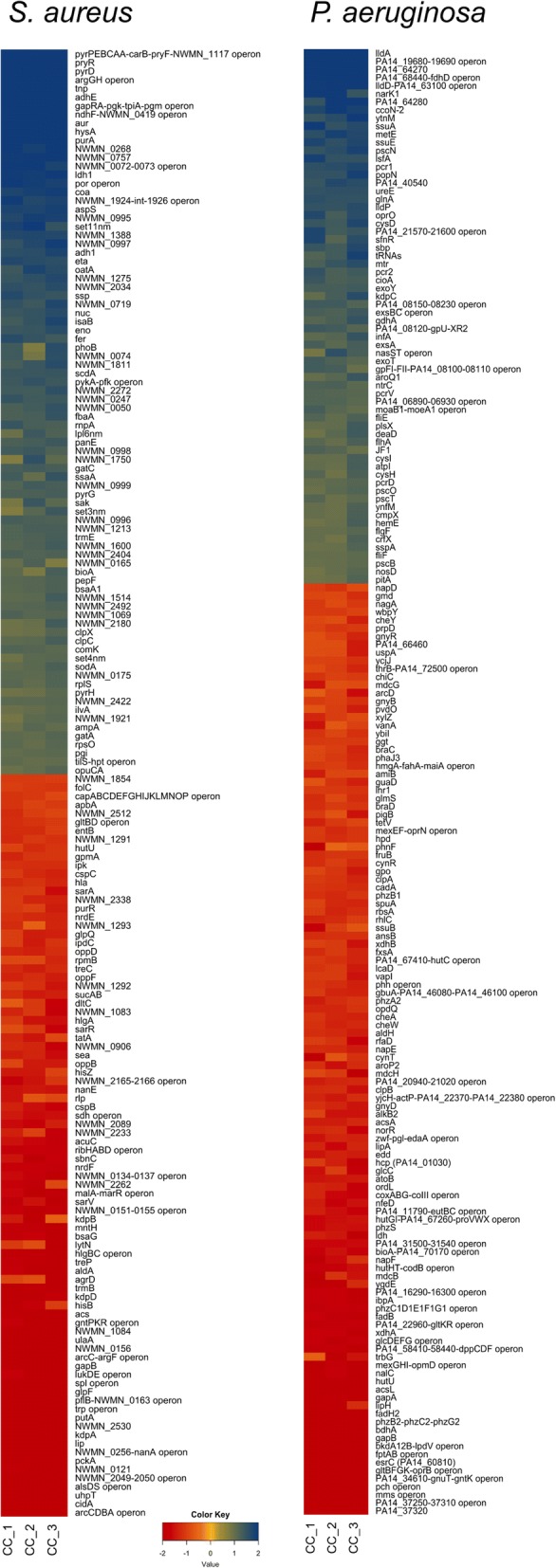
Heat map of differentially expressed genes in *P. aeruginosa* (left) and *S. aureus* (right) in co-cultures compared to mono-cultures expressed in log_2_ base. Only genes showing a log2 ratio ≤ −1 and ≥ 1, are shown. When applicable, genes were clustered in operons

**Fig. 3:**
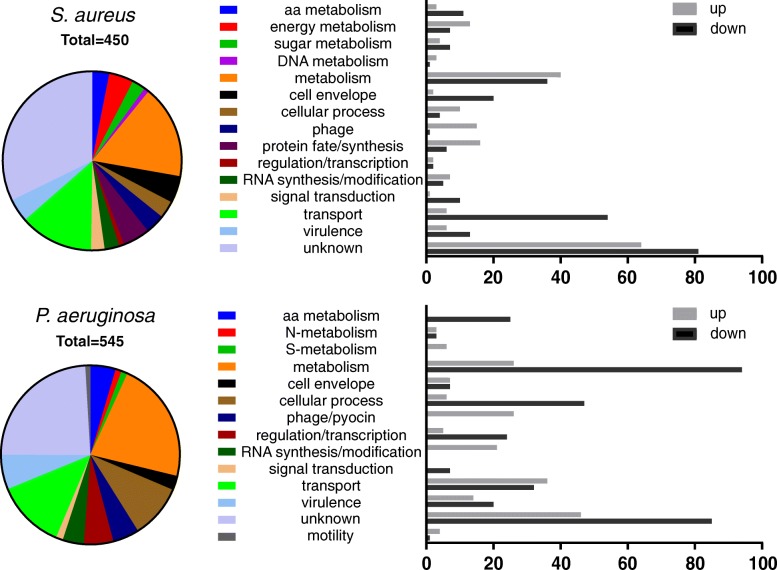
Functional classification of genes and their change in expression (> 2-fold up or > 2-fold down) compared to the mono-culture. The x-axis shows the absolute number of genes differentially regulated in the presence of competitor versus the corresponding mono-culture

To study the respective responses of *P. aeruginosa* and *S. aureus* at the very early stage of interaction, we compared the gene expression profile of both bacteria after 3 h of co-culture versus mono-culture. At 3 h the CFU ratio of *P. aeruginosa* and *S. aureus* was approximately 50% and comparable to the initial ratio at *t* = 0 (Fig. 1) suggesting that effects of potential competition had not translated into changes in population dynamics. *S. aureus* CFU started to decrease after 4 h (data not shown). Despite the conserved population structure after 3 h of co-culture, the transcriptome in each bacterium was significantly affected by the presence of the other one, when compared to the mono-culture transcriptome. Table 1 shows the significantly differentially expressed genes (false discovery rate (FDR) 5%). 1726 and 1718 genes were down and up-regulated, respectively, in *P. aeruginosa* co-culture versus mono-culture, while 761 and 730 were down and up-regulated, respectively, when *S. aureus* was growing in co-culture compared to the mono-culture. The proportion of genes that showed a more than 2-fold change in expression (log2 ratio ≥ 1 or ≤ − 1), accounted for 9.4% of the *P. aeruginosa* genome (545 genes) and 18.6% of the *S. aureus* genome (450 genes). Among these, 181 genes (33.2%) and 189 genes (42.1%) were up-regulated, whereas 364 genes (66.8%) and 261 genes (47.9%) were down-regulated in *P. aeruginosa* and *S. aureus*, respectively (Table 1, Fig. 2, Additional file 1: Figure S1). The functional classification of the differentially regulated genes showed that metabolism and transport were the most affected classes. Interestingly the proportions of these categories were similar in both organisms with 28.8% (*P. aeruginosa*) and 27.8% (*S. aureus*) for metabolism and 12.5% (*P. aeruginosa*) and 13.3% (*S. aureus*) for transport functions (Fig. 3). RNAseq data were validated by quantitative real-time PCR for six genes per bacterium (3 up- and 3 down-regulated genes), showing a linear correlation with the transcriptome data (Additional file 2: Figure S2).

**Table 1:** Differentially expressed genes in co-culture vs mono-culture (FDR < 5%)

Condition	Up-regulated	Down-regulated	log_2_FC > 1	log_2_FC < −1
CC vs PM	1726	1718	181	364
CC vs SM	761	730	189	261

*CC* co-culture, *PM P. aeruginosa* mono-culture, *SM S. aureus* mono-culture

*FDR* false discovery rate, *FC* fold-change

### Oxygen limitation occurs early in co-culture

**Fig. 4:**
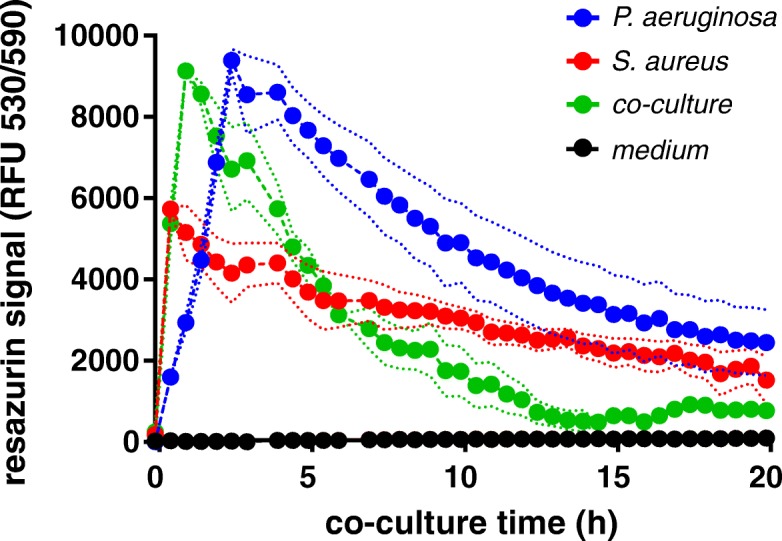
Measurement of oxygen status by resazurin. Oxygen is maximal 1 to 3 h post-inoculation. While in the *S. aureus* mono-culture, oxgen availability decreases slowly, oxygen depletion occurs faster in co-culture with *P. aeruginosa* and drops to microaerophilic conditions after 13 h. Shown is the average and the standard deviation (dotted lines) of three independently performed biological replicates. RFU, relative fluorescent units (excitation/emission wavelengths)

After 3 h of incubation, the transcriptome data was consistent with a switch from aerobic towards anoxic conditions. Reflected mainly by a 3 to 4-fold reduction in expression of the four cytochrome C oxidase genes (*coxA*-*coxB*-PA14_01310-*coIII*) and the apparent accumulation of nitrate in the cytosol that *P. aeruginosa* can use as an electron acceptor under anoxic conditions (Table 3). This is achieved by induction of *narK1*, encoding a cytoplasmic membrane antiporter that exports nitrite in exchange for nitrate [27], as well as *nasS* encoding a nitrate importer. In contrast, *napF* and *napE,* encoding subunits of the periplasmic nitrate reductase were down-regulated in *P. aeruginosa*. These observations indicate that nitrite production/import is inhibited thereby increasing the cytosolic nitrate concentration, which suggests a preferential use of the inner membrane-associated nitrate reductase (Nar), rather than the periplasmic nitrate reductase (Nap). To assess the oxygen status under the experimental setup, we added resazurin to mono and co-cultures, and followed fluorescence emission. Irreversible reduction of the non-fluorescent resazurin (blue) to the fluorescent resorufin (pink) occurred within less than 1 h for the *S. aureus* mono-culture and the co-culture (Fig. 4). Reduction was slower with the *P. aeruginosa* mono-culture reaching a maximal fluorescence after 2 h. Oxygen availability then decreased steadily in mono and co-cultures, but reached an almost undetectable fluorescence level after 14 h only in the co-culture. *P. aeruginosa* preferentially uses O_2_ as an electron acceptor and hence more rapidly depletes the available oxygen in the medium.

### Increased expression of fermentation pathways in *S. aureus* and lactate utilization genes in *P. aeruginosa*

**Fig. 5:**
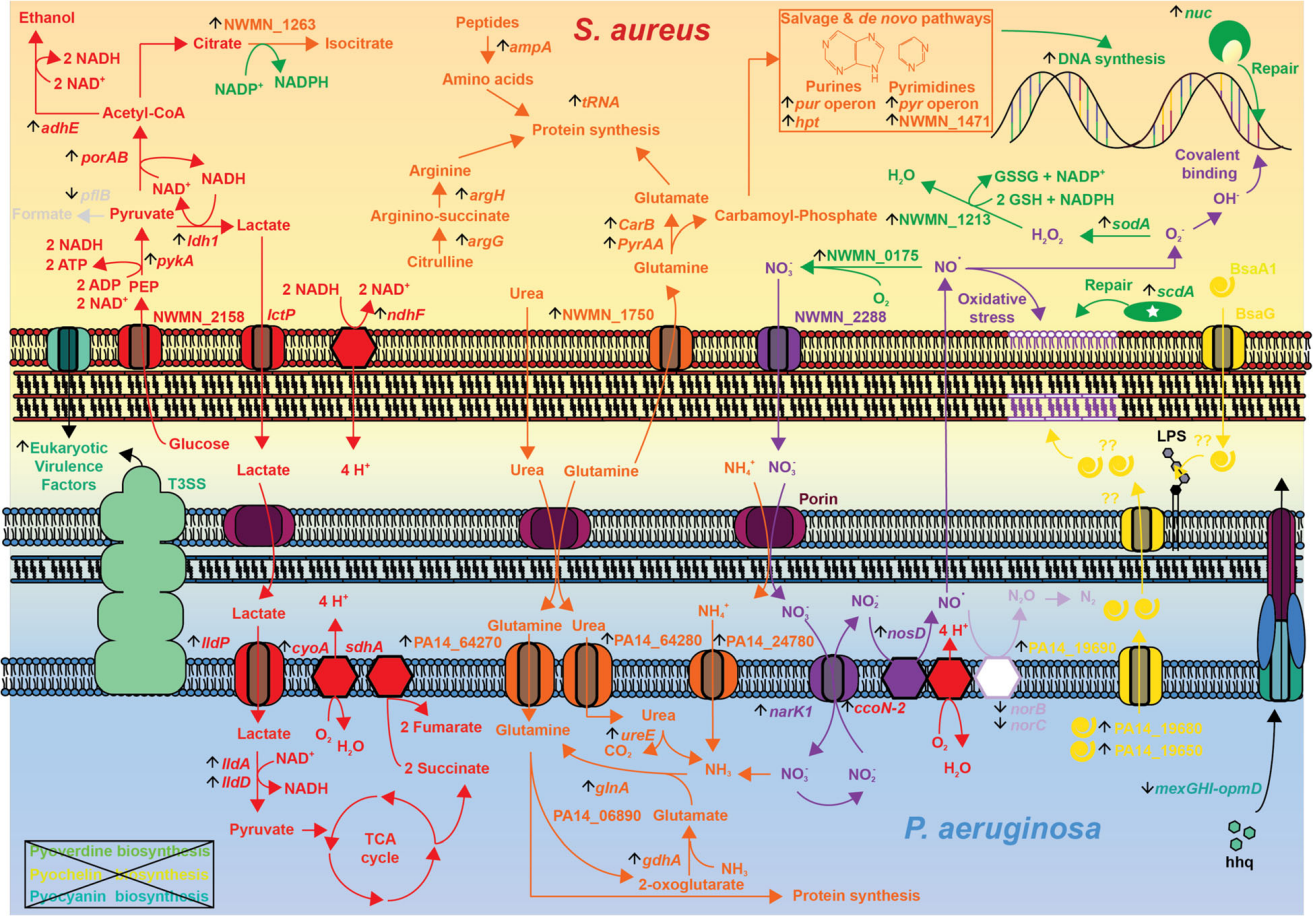
Schematic overview of the main reponses deduced by transcriptome analysis of *P. aerugiosa* and *S. aureus* after 3 h of co-culture. Vertical black arrows indicate genes up- or down-regulated in the presence of the competitor. Details are found in the text. Fold changes are reported in Tables 2 and 3 and in Additional file 3: Table S1 and Additional file 4: Table S2. Pathways involved in carbon and nitrogen metabolism affected in both organisms during the co-culture are shown in red and orange, respectively. Pathways affected in the co-culture and involved in the generation or in the response to oxidative stress are shown in green in *S. aureus* and in violet in *P. aeruginosa*. Induction of the holin/anti-holin like proteins (PA14_19680, PA14_19691) in *P. aeruginosa* are indicated in yellow (bottom right). After 3 h of co-culture, the synthesis of the siderophores pyoverdin and pyochelin as well as of the phenazines was strongly reduced in *P. aeruginosa* in response to *S. aureus* (box in the bottom left corner)

In response to the decreased oxygen availability, we noticed an increased expression of genes encoding enzymes for fermentation of glucose into pyruvate, encoded by the *gap-pgk-tpi-pgm-eno* operon in *S. aureus* (Table 2 and Additional file 3: Table S1). Genes for enzymes involved in conversion of pyruvate into formate (*pflB*) and acetoin (*alsS, alsD)* were repressed 6 and 10-fold, respectively. In contrast, the lactate dehydrogenase gene *ldh1* and genes of the AdhE pathway (*porAB, adhE, adh1*) were upregulated 3 to 7-fold, indicating that *S. aureus* preferentially converted pyruvate into secreted lactate and ethanol. Hence, in our experimental setup, *S. aureus* mainly produced energy from glycolysis and fermentation of pyruvate into lactate (Table 2 and Additional file 3: Table S1). In line with these observations, the most upregulated gene of *P. aeruginosa* in response to *S. aureus,* was the membrane bound L-lactate dehydrogenase LldA (14-fold increase). Concomitant increase in expression (3 to 4-fold) of the second lactate dehydrogenase operon including *lldD*, the adjacent *lldP* lactate permease gene (3-fold) and PA14_63100 coding for a cytochrome type lactate dehydrogenase (Table 3 and Additional file 4: Table S2), strongly suggests that *P. aeruginosa* takes up lactate secreted by *S. aureus* to use it as a preferential carbon and energy source (Fig. 5).

**Table 2:** Selection of differentially expressed genes in *S. aureus* in co-culture versus mono-culture

gene	FC^a^	annotation	functional category
Up-regulated genes			
*pyrP*	255.62	uracil permease	metabolism
*pyrC*	126.35	dihydroorotase	metabolism
*pyrB*	94.35	aspartate transcarbamoylase	metabolism
*pyrA*	94.34	Carbamoyl-phosphate synthase small chain	metabolism
*carB*	56.66	Carbamoyl-phosphate synthase large chain	metabolism
*pyrR*	54.32	Bifunctional protein PyrR	metabolism
*pyrF*	37.64	Orotidine 5′-phosphate decarboxylase	metabolism
*pyrE*	29.69	Orotate phosphoribosyltransferase	metabolism
*pyrD*	24.95	Dihydroorotate dehydrogenase (quinone)	metabolism
*argG*	15.30	Argininosuccinate synthase	aa metabolism
*argH*	12.49	Argininosuccinate lyase	aa metabolism
*adhE*	7.64	alcohol dehydrogenase	metabolism
NWMN_1896	6.34	phage major capsid protein NM1	phage
*gapR*	6.00	glycolytic gap-pgk-tpi-pgm-eno operon	metabolism
*ndhF*	5.88	NADH:menaquinone oxidoreductase (subunit 5)	metabolism
*pgk*	5.81	glycolytic gap-pgk-tpi-pgm-eno operon	metabolism
*tpiA*	5.67	glycolytic gap-pgk-tpi-pgm-eno operon	metabolism
*gapA*	5.57	glycolytic gap-pgk-tpi-pgm-eno operon	metabolism
*pgm*	5.49	glycolytic gap-pgk-tpi-pgm-eno operon	metabolism
*porA*	5.18	Pyruvate flavodoxin ferredoxin oxidoreductase	metabolism
*purA*	4.42	Adenylosuccinate synthetase	meatbolism
NWMN_0997	3.26	phage NM2	phage
*adh1*	3.17	Alcohol dehydrogenase	metabolism
*scdA*	2.72	Iron-sulfur cluster repair protein ScdA	metabolism
NWMN_1213	2.37	Glutathione peroxidase	metabolism
*sodA*	2.18	Superoxide dismutase	metabolism
*hpt*	2.01	Hypoxanthine phosphoribosyltransferase	metabolism
Down-regulated genes			
*arcB*	−35.81	Ornithine carbamoyltransferase	aa metabolism
*arcA*	−26.57	Arginine deiminase	aa metabolism
*arcD*	−22.97	Arginine/ornithine antiporter	aa metabolism
*cidA*	−16.67	Holin-like protein CidA	cell envelope
*alsD*	−14.89	Alpha-acetolactate decarboxylase	metabolism
*alsS*	−10.50	Alpha-acetolactate synthase	metabolism
*splD*	−10.50	Serine protease SplD	virulence
*putA*	−6.94	Proline dehygrogenase	metabolism
*pflB*	−5.92	Formate acetyltransferase	metabolism
*capJ*	−5.60	Capsular polysaccharide biosynthesis	cell envelope
*capI*	−5.59	Capsular polysaccharide biosynthesis	cell envelope
*trpD*	−5.57	Anthranilate phosphoribosyltransferase	aa metabolism
*lukE*	−5.52	leukotoxin LukE	virulence
*glpF*	−5.47	glycerol uptake facilitator	transport
*capN*	−5.46	Capsular polysaccharide biosynthesis	cell envelope
*hisB*	−5.39	Imidazoleglycerol-phosphate dehydratas	metabolism
*trpE*	−5.31	Anthranilate synthase component I	aa metabolism
*capL*	−5.25	Capsular polysaccharide biosynthesis	cell envelope
*capO*	−4.94	Capsular polysaccharide biosynthesis	cell envelope
*splA*	−3.91	Serine protease SplA	virulence
*splB*	−3.44	Serine protease SplB	virulence
*sarV*	−3.47	Staphylococcal accessory regulator	signal transduction
*murP*	−3.04	sucrose-specific PTS transporter IIBC component	transport
*lytN*	−2.35	cell wall hydrolase	cell envelope
*sarA*	−2.30	Global transcriptional regulator	signal transduction
*sarR*	−2.20	Staphylococcal accessory regulator	signal transduction
*purR*	−2.18	Pur operon repressor	metabolism
*gltD*	−2.11	Glutamate synthase, small subunit	N-metabolism
*gltB*	−2.10	Glutamate synthase, large subunit	N-metabolism

^a^*FC* fold change; values are the average of three replicates after normalization

**Table 3:** Selection of differentially expressed genes in *P. aeruginosa* in co-culture versus mono-culture

gene	FC ^a^	annotation	functional category
Up-regulated genes			
*lldA*	14.13	L-lactate dehydrogenase	metabolism
PA14_19690	7.77	CidB, LrgB anti-holin protein	cell envelope
PA14_19680	6.51	CidA, LrgA holin like protein	cell envelope
*fdnH*	5.55	nitrate inducible formate DH accessory protein	metabolism
PA14_64270	5.08	Leu/Ile/Val-binding protein family signature	transport
PA14_55631	5.05	23S ribosomal RNA	RNA synthesis/modif.
PA14_62060	5.05	23S ribosomal RNA	RNA synthesis/modif.
PA14_61830	4.88	tRNA-Met	RNA synthesis/modif.
PA14_63100	4.38	cytochrome type D-lactate DH (4Fe-4S)	metabolism
*narK1*	3.95	nitrite extrusion protein I	transport
PA14_24780	3.87	Amoonium transporter	transport
*lldD*	3.64	L-lactate dehydrogenase	metabolism
PA14_60150	3.26	tRNA-Lys	RNA synthesis/modif.
*popN*	3.13	type III secretion system	virulence
PA14_08670	3.01	tRNA-Thr	RNA synthesis/modif.
PA14_08660	2.96	tRNA-Gly	RNA synthesis/modif.
*lldP*	2.94	L-lactate permease	transport
*ureE*	2.93	urease accessory protein UreE	metabolism
*glnA*	2.93	glutamine synthetase	N-metabolism
*mtr*	2.74	Tryptophan permease	transport
PA14_36220	2.61	Amino acid permease	transport
*exsB*	2.56	TTSS regulator	virulence
PA14_08210	2.52	F-pyocin	Phage/pyocin
*gdhA*	2.49	glutamate dehydrogenase	N-metabolism
PA14_06890	2.36	pyruvate kinase pyridoxal phosphate	metabolism
PA14_06930	2.32	glutamine amidotransferase	metabolism
PA14_08070	2.30	R-pyocin, phage tail protein	Phage/pyocin
PA14_06920	2.04	class III pyridoxal phosphate aminotransferase	metabolism
Down-regulated genes			
PA14_37310	−20.35	allophanate hydrolase subunit II putative	metabolism
PA14_37290	−18.80	allophanate hydrolase subunit I putative	metabolism
PA14_37270	−18.65	LamB/YcsF, carbohydrate/lactam utilization	metabolism
*mmsB*	−16.48	3-hydroxyisobutyrate dehydrogenase	aa metabolism
*mmsA*	−15.68	methylmalonate-semialdehyde dehydrogenase	aa metabolism
PA14_37260	−15.13	OpdO, lactam/pyroglutamate uptake	transport
*pchB*	−14.79	pyochelin synthesis	virulence
PA14_37250	−12.58	MFS transporter	transport
*bkdA1*	−11.87	2-oxoisovalerate dehydrogenase subunit alpha	aa metabolism
*gnuT*	−11.53	gluconate permease	transport
*pchC*	−11.32	pyochelin synthesis	virulence
*pchG*	−10.48	pyochelin synthesis	virulence
*pchA*	−9.97	pyochelin synthesis	virulence
*pchF*	−9.56	pyochelin synthesis	virulence
PA14_23010	−9.47	GltK, ATP-binding component of ABC transporter	transport
PA14_23000	−9.37	permease of ABC sugar transporter	transport
PA14_22990	−9.19	permease of ABC sugar transporter	transport
PA14_23030	−8.63	OprB, Glucose/carbohydrate porin	transport
*bkdA2*	−8.05	2-oxoisovalerate dehydrogenase subunit beta	aa metabolism
*bdhA*	−5.81	3-hydroxybutyrate dehydrogenase	metabolism
*bkdB*	−4.93	branched-chain alpha-keto acid dehydrogenase	metabolism
*lpdV*	−3.58	dihydrolipoamide dehydrogenase	metabolism
*fptA*	−4.85	pyochelin receptor	virulence
*mexG*	−4.32	RND efflux pump	transport
*mexH*	−4.12	RND efflux pump	transport
*opmD*	−3.49	RND efflux pump	transport
*mexI*	−3.43	RND efflux pump	transport
*coxA*	−3.90	Cytochrome c oxidase subunit I	energy metabolism
*coxB*	−2.98	Cytochrome c oxidase subunit II	energy metabolism
*PA14_01310*	−2.64	Cytochrome c oxidase assembly protein	energy metabolism
*coIII*	−2.15	Cytochrome c oxidase subunit III	energy metabolism
*napF*	−2.97	periplasmic nitrate reducatse	N-metabolism
*napE*	−2.45	periplasmic nitrate reducatse	N-metabolism

^a^*FC* fold change; values are the average of three replicates after normalization

### Co-culture induced starvation and competition for glutamine as nitrogen and energy source

After 3 h of co-culture, we detected signs of nutrient starvation and potential responses in both organisms. Nitrogen starvation by *P. aeruginosa* was highlighted by up-regulation of the master nitrogen regulator gene *ntrC*, which responds to limitation of N-sources, including urea, purines, amino acids and in particular glutamine [28]. In co-culture, increased expression of *ntrC* coincided with increased expression of *gdhA, glnA* and *ureE*, which respectively code for glutamate dehydrogenase, glutamine synthase, and a urease accessory factor, all involved in nitrogen assimilation (Table 3). In addition, the expression of the entire PA14_06890-PA14_06930 operon, which encodes putative glutamine amido/aminotransferases, was increased in the presence of *S. aureus*. Interestingly, we also observed a response compatible with nitrogen starvation in *S. aureus*, since both *gltB* and *gltD*, coding for the large and small subunit of glutamate synthase that converts glutamine to glutamate, were significantly decreased during co-culture. Moreover, both bacteria seemed to acquire extracellular N-sources as reflected by the increased expression of NWMN_1750 in *S. aureus*, encoding a glutamine binding protein, and PA14_24780 in *P. aeruginosa*, an ammonium transporter. Taken together, these findings indicate that co-culture rapidly induces conditions of nitrogen starvation in both bacteria, where glutamine seems to play a central role (Fig. 5).

### Increased pyrimidine/purine synthesis and salvage pathways in *S. aureus*

Pyrimidines and purines are key components for DNA/RNA synthesis, cell-signaling ((p)ppGpp, c-di-GMP) and energy production (ATP, GTP). The most highly induced gene (255-fold) in *S. aureus* in response to *P. aeruginosa*, encodes the uracil transporter PyrP, followed by the pyrimidine de novo synthesis operon (*pyrPEBCAA-carB-pyrF-*NWMN_117) (126 to 25-fold) (Table 2, Fig. 2 and Additional file 3: Table S1), encoding all enzymes required for the conversion of glutamine to uridine mono-phosphate (UMP). Furthermore, we observed decreased expression of *purR*, encoding the *pur* operon repressor, with concomitant increased expression of *purA* of the de novo purine synthesis pathway. Together with the upregulation of *hpt,* encoding the hypoxanthine-guanine phosphoribosyl-transferase of the purine salvage pathway, this should result in increased intracellular purine levels. Taken together our observations indicate that activation of pyrimidine and purine synthesis via de novo and salvage pathways represent the most dramatic early response of *S. aureus* to the presence of *P. aeruginosa* (Fig. 5).

### Amino acid starvation responses and decreased carbohydrate utilization

*S. aureus* strains are auxotrophic for arginine, proline, valine, cysteine and leucine [29], despite the fact that *S. aureus* possesses the corresponding biosynthetic pathways [30]. It was therefore surprising to find a very strong response in genes involved in arginine biosynthesis pathways. Genes a*rcA-arcB-arcC* of the arginine catabolic operon were downregulated 33 to 7-fold, while *argG* and *argH*, encoding enzymes for conversion of citrulline to arginine were up-regulated (13 to 15-fold). This suggests a strong tendency to maintain or increase cytosolic arginine levels. Furthermore, the *putA* gene involved in an alternative arginine biosynthesis pathway was downregulated 7-fold (Table 2), probably to maintain sufficient proline concentrations [30]. In *P. aeruginosa*, a prototrophic organism, an increase in *sspA*, coding for the stringent starvation protein A, is indicative of amino acid shortage. In agreement with this, genes for branched chain amino acid (leucine, valine, isoleucine) transporters (PA14_64270, PA14_64280) and amino acid permeases (*mtr*, PA14_36220) were up-regulated 3 to 7-fold. Furthermore *P. aeruginosa* strongly repressed branched-chain amino acid degradation as illustrated by a 5 to 16-fold decrease in expression of the *lpdV*-*bkdB-bdhA-bdkA1 and mmsA-mmsB* operons (Table 3 and Additional file 4: Table S2). Since *S. aureus* requires leucine and valine, this response could further increase amino acid starvation in *S. aureus*.

Genes for sugar and carbohydrate metabolism in *P. aeruginosa* were among the most strongly downregulated genes in the presence of *S. aureus*. These include the operon containing genes for the glucose/carbohydrate specific OprB porin and the adjacent ABC-transporters (PA14_22990 and PA14_23000) (10-fold decrease), the operon containing the OpdO porin and the co-transcribed ABC-transporter PA14_37250 (15 to 20-fold decrease), involved in pyroglutamate/lactam uptake as well as the gluconate permease gene *gnuT*. The data clearly illustrate an effort in both organisms to maintain or increase intracellular amino acid pools, while downregulating sugar and carbohydrate uptake and metabolism.

Another indicator of amino acid starvation is the accumulation of probably uncharged tRNAs, observed in *P. aeruginosa* (Table 3 and Additional file 4: Table S2). Uncharged tRNAs bind the ribosome and stimulate the synthesis of the alarmones ppGpp and pppGppp by the RelA/SpoT enzymes, signaling nutrient starvation stress. In *S. aureus* we did not observe a similar increase in tRNA pools, but observed up-regulation of tRNA aminoacylation and rRNA genes (Additional file 3: Table S1).

### Antioxidant response by *S. aureus*

The transcriptional profile of *S. aureus* indicated signs of oxidative stress as illustrated by the increased expression of several genes encoding antioxidant proteins and more strikingly, NWMN_0175, encoding an NO dioxygenase (2-fold) [31], catalyzing NO detoxification by conversion into nitrate as well as *sodA* (2-fold) encoding the superoxide dismutase, which detoxifies superoxide (O_2_^−^) into dioxygen (O_2_), hydrogen peroxide (H_2_O_2_) and H_2_O [32]. Expression of NWMN_1213, encoding a putative glutathione peroxidase, was also increased (2.4-fold), which would allow further detoxification of the produced hydrogen peroxide produced by turning it into H_2_O. Finally, expression of *scdA*, encoding a membrane-repairing protein induced by oxidative stress in cell membranes [33, 34] was increased (2.7-fold). Taken together, our results reveal strong evidence for increased oxidative stress sensed by *S. aureus*, which could result from acetate-induced ROS production under excess glucose concentrations or be induced by a metabolite [35, 36] (Fig. 5).

### Co-culture decreases virulence in *S. aureus* and *P. aeruginosa*

*P. aeruginosa* possesses several multidrug efflux pumps to protect itself from harmful molecules including antibiotics [37]. The expression of one of the resistance-nodulation-division (RND) type efflux pumps, encoded by the *mexGHI-opmD* operon was decreased 4 to 5-fold in co-culture (Table 3). The MexGHI-OpmD pump has been linked to vanadium resistance [38] and has recently been shown to transport 5-methylphenazine-1-carboxylate, a toxic intermediate in phenazine synthesis [39]. This is in agreement with a 2 to 6-fold downregulation of genes belonging to the two phenazine biosynthesis operons. Unexpectedly, expression of the entire pyochelin synthesis operon (*pchDCBA, pchEFG*) and of the FptA pyochelin receptor gene was also downregulated 6 to 15-fold, which would indicate that iron was not limiting at this stage of growth. In contrast, expression of several type 3 secretion system (T3SS) genes (*exsB*-*C*, *pscN*, *popN*, *pcr1* and *pcr2*) was increased 3-fold, despite the obvious absence of eukaryotic cells (Table 3 and Additional file 4: Table S2).

Similarly, *S. aureus* decreased expression of many virulence factors in response to *P. aeruginosa*. Among them, the global (*sarA*) and accessory (*sarR*, *sarV*) virulence regulator genes (2 to 3-fold) (Additional file 3: Table S1), the capsular polysaccharide operon *capA-capP* (2 to 8-fold), as well as leukocidine and the secreted serine protease genes (*splA* to *splF*) (Table 2 and Additional file 3: Table S1). Since many of these virulence factors are costly to produce and target host cells, their downregulation should save resources and energy required for metabolic processes. No change in the expression of antibiotic resistance genes was observed.

### Opposite responses in cell wall lysis in *S. aureus* and *P. aeruginosa*

The CidA-CidB and LrgA/LrgB proteins constitute holin/anti-holin systems in *S. aureus* that regulate cell lysis in a programmed-cell-death manner and are required for biofilm formation through release of eDNA [40, 41]. Several genes that are involved in programmed cell lysis were significantly down-regulated in response to *P. aeruginosa*. In particular, the holin protein gene *cidA* was among the most affected genes (17-fold decrease) in co-culture. Furthermore, expression of the *lytN* cell wall hydrolase gene and the peptidoglycan degrading enzymes encoded in the NWMN_0134-*murQ*-*murP*-*murR* operon were also repressed in presence of *P. aeruginosa* (Table 2). In contrast, the so far uncharacterized *cidA* (PA14_16690, PA3431) and *cidB* (PA16680, PA3432) orthologues, present in *P. aeruginosa* were among the three most up-regulated genes in response to *S. aureus* (Table 3 and Additional file 4: Table S2). These holin/anti-holin systems regulate the access of endolysins to the periplasm and therefore control cell wall degradation and cell death.

### Induction of phage and pyocin operons

Intriguingly and in contrast to the above observations, was the induction of all four prophage loci of the *S. aureus* Newman strain, including the anti-repressor genes of prophages NM2 and NM4, and of structural phage genes from the NM1 (3 to 9-fold induction) and NM3 prophage operons (Table 2 and Additional file 3: Table S1). Similarly, the R- and F-pyocin genes in *P. aeruginosa* were induced in response to *S. aureus* (Additional file 4: Table S2). R- and F- as well as S-pyocins are widely distributed in *P. aeruginosa* strains and are induced in response to mitomycin C, oxidatvive stress (H_2_O_2_), as well as ciprofloxacin [42]. Hence, the presence of the co-colonizing organism is recognized as a stress signal and likely causes lysis of a small fraction of the bacterial population.

## Discussion

We attempted to analyze the early responses of *P. aeruginosa* and *S. aureus* when co-cultured for a short period (3 h) by transcriptomic analysis. Previous reports have focused on biofilm co-cultures in Brain Heart Infusion medium [43] and on growth on a CF-lung epithelium cell line performed in cell culture medium (MEM + 2 mM glutamine) [44]. Despite the very different culture conditions, we noticed some overlapping responses described in these two studies with our in vitro co-culture model but also identified some specific responses. A notable difference with the biofilm study was the very low number of genes (16) differentially regulated in *P. aeruginosa* [43], compared to the > 500 genes in our study using planktonic cells. This might be due to the different experimental setups, considering that metabolism is reduced during biofilm growth, while in our conditions both bacterial strains were in exponential growth phase requiring maximal metabolic activity.

It is therefore not surprising that the strongest responses measured by gene expression involved metabolic pathways in both bacteria. In particular, we found that *S. aureus* likely fermented glucose into lactate. As a logical consequence, we observed in *P. aeruginosa,* increased expression of lactate transporters, and pathways for conversion of lactate into pyruvate, which can subsequently fuel the TCA cycle. A similar response was observed by a previous transcriptome study on *S. aureus*-*P. aeruginosa* co-culture performed on a CF-respiratory epithelium, which showed increased lactate production in *S. aureus* in response to *P. aeruginosa* [44]. Moreover, the authors showed that *P. aeruginosa* preferentially uses lactate over glucose when both C-sources are available. Our data obtained after only 3 h of co-culture, and in the absence of epithelial cells, showed down-regulation of *pflB*, *aldA* and *alsD-alsS* genes (acetoin, formate synthesis), and upregulation of *ldh* (lactate) and *adh* (ethanol) genes in *S. aureus*, which is in agreement with the study of Filkins et al. showing similar changes after 16 h of co-culture [44]. This indicates that lactate production is an early co-culture response and is independent of the presence of host epithelial cells. Additionally, we observed upregulation of lactate utilization (*lldA*, *lldD*) and uptake (PA14_63100) genes in *P. aeruginosa*, corroborating their metabolic data.

After 3 h of co-culture the transcriptome showed signs of a transition from aerobic to anaerobic conditions. As shown by the resazurin experiments, oxygen availability decreased more rapidly in the co-culture compared to the mono-cultures. Also, the threshold levels for a switch from aerobic to microaerophilic conditions might differ between the two organisms. This might explain that some of the anaerobically induced pathways were detected in *P. aeruginosa* but not in *S. aureus*. Considering that the CF-lung represents an oxygen scarce environment [45], the observed metabolic changes observed between *P. aeruginosa* and *S. aureus* in vitro might be of particular importance also under these in vivo conditions [46].

Our data further suggest that the co-culture conditions lead to a state of nitrogen starvation in both bacteria. N-sources play a key role in all cellular anabolic processes such as protein and nucleotide biosynthesis. Purines and pyrimidines are constituents of both DNA and RNA, and purines are specifically important for energy metabolism (ATP, NADH, coenzyme A) and as secondary messengers (c-di-GMP, cAMP, (p)ppGpp). In this context, glutamine represents a high-yield nitrogen and carbon source for microorganisms when processed by aminotransferases [47]. Our data indicate that the strongest response in *S. aureus* included de novo synthesis and activation of the salvage pathway for both purines and pyrimidines. In addition, increased expression of tRNAs (Table 3 and Additional file 4: Table S2) coupled with signs of nitrogen starvation point to a state of increased protein synthesis in both bacteria in co-culture. We therefore believe that nitrogen sensing represents a specific early response during competition between these two organisms.

Detrimental interactions between different bacterial species may involve contact-independent inhibition, via synthesis of antimicrobial molecules, or through contact-dependent inhibition [14, 18]. In agreement with this hypothesis, we observed increased expression of numerous antioxidant responses in *S. aureus* and in particular the NO-dioxygenase, which detoxifies NO [31]. However, none of the known anti-staphylococcal products (LasA protease, HQNO, pyocyanin) were upregulated under our experimental conditions in *P. aeruginosa*. Since these products are quorum-sensing (QS)-controlled, their expression is likely to be induced during late exponential growth phase and independently of a competitor.

Interestingly, both organisms showed potential signs of cell lysis during co-culture, as suggested by induction of R- and F-pyocin synthesis genes in *P. aeruginosa* (Table 2) and several prophage genes (NM1 to NM4) in *S. aureus* (Table 3). Cell lysis may have occurred likely in a small sub-population of the culture in response to nutrient limitation or direct cell damage, and could allow the bacteria to detect the presence of a competitior, a mechanism known as competition sensing [48]. Prophage induction in *S. aureus* also occurs in the presence of hydrogen peroxide, produced for instance by *Streptococcus pneumonia* strains [49]. *P. aeruginosa* does not produce hydrogen peroxide but ROS or RNS species generated during the initial aerobic growth phase might induce this response in *S. aureus*.

Co-culture between *P. aeruginosa* and *S. aureus* also affected the expression of virulence factors in both bacteria. In order to colonize and survive within the host, bacteria express virulence factors, which allow them to adhere to host tissue and to get access to nutrients [50]. However, in this study *S. aureus* responded to the co-culture by decreasing the secretion of capsular polysaccharide, leukotoxins, general virulence regulators, serine proteases, none of which are known to have an effect on other bacteria. Similarly, *P. aeruginosa* showed decreased expression of biosynthesis pathways for the secreted virulence factors pyocyanin and pyochelin. The decreased expression of the RND multidrug efflux pump MexGHI-OpmD is consistent with these data [38]. Indeed, mutants in *mexI* and *opmD* are unable to produce the Pseudomonas Quinolone Signal (PQS, 2-heptyl-3-hydroxy-4-quinolone) and N-(3-oxododecanoyl)-L-homoserine lactone (3-oxo-C12-HSL), while showing a marked decreased production in N-butanoyl-L-homoserine lactone levels (C4-HSL). These defects have been reported to cause significant reduction in the production of QS-dependent virulence factors including elastase, rhamnolipids, pyocyanin, pyoverdin and significantly reduce swarming motility [51]. These observations suggest that in the absence of host cells, both organism decrease virulence factor production.

## Conclusions

Our dual-transcriptome analysis on *S. aureus* and *P. aeruginosa* co-culture reveals an intricate pattern of genetic adaptations during the initial encounter between these bacterial pathogens (Fig. 5). The responses are dominated by metabolic changes, since the largest number of differentially expressed genes belong to the functional classes “metabolism” and “transport”. In particular, our data confirm the previous observation that *P. aeruginosa* drives *S. aureus* into fermentation, allowing the former organism to use lactate, produced by the latter organism, as a C-source [44]. Both organisms show signs of nitrogen starvation that likely induce purine and pyrimidine synthesis pathways in *S. aureus*. We found no evidence for induction of anti-staphylococcal pathways in *P. aeruginosa*, suggesting that resource competition rather than direct (interference) competition prevail during the early stages of co-culture. Both organisms seem to sense stress caused by nutrient deprivation, ROS-mediated attack or direct cell damage. These stressor may induce cell lysis in a subpopulation of the culture caused by induction of pyocins in *P. aeruginosa* and prophages in *S. aureus*.

## Methods

### Strains and culture conditions

Bacterial strains used in the study are *P. aeruginosa* strain PA14 [52] and *S. aureus* Newman [53]. Bacteria were inoculated from − 80 °C glycerol stocks and grown overnight on M14 agar plates. M14 medium is based on M9 salts (Na_2_HPO_4_ 6 g/L; KH_2_PO_4_ 3 g/L; NaCl 0.5 g/L; NH_4_Cl 1 g/L) [54] supplemented with 10 g/L casamino acids (BD™, United States), magnesium sulfate (MgSO_4_) 1 mM, 2 mg/L thiamine (vitamin B1), 2 mg/L niacin (vitamin B3), 2 mg/L calcium pantothenate (vitamin B5), 0.1 mg/L biotin (vitamin B9) and 2 g/L glucose (11 mM). In this medium *S. aureus* and *P. aeruginosa* displayed similar growth rates when incubated at 37 °C in a microtiter plate [55].

### Short-term competition assay

The short-term competition assay was performed as illustrated in Fig. 1. *P. aeruginosa* PA14 and *S. aureus* Newman were grown overnight in M14 medium and diluted separately in this medium to an optical density (OD_600_) = 1.0. Ten μL from these bacterial suspensions were added to a microtiter plate (TPP®, Switzerland) containing 190 μL of M14 medium per well. One row was inoculated with *P. aeruginosa* and one row with *S. aureus*, only. The microtiter plate was incubated under static conditions in a Multi-Mode plate reader (Synergy H1, BioTek, USA) for 3 h. OD_600_ was measured at regular intervals following 1 min shaking. After the 3 h pre-incubation, co-cultures were prepared by mixing 100 μL of each mono-culture within an empty well obtaining a final volume of 200 μL. The remaining volume was discarded. The wells for the mono-culture were left unchanged (no addition of fresh medium). Rows A and H contained only medium to avoid evaporation at the plate edges and as a control for contamination. After 3 h of incubation, four identical wells of 200 μL were pooled together, representing one replicate. Three replicate samples were labelled as *P. aeruginosa* mono-cultures (PM1, PM2, and PM3), *S. aureus* mono-cultures (SM1, SM2, and SM3) and co-cultures (CC1, CC2, and CC3).

Plate countings were performed after the 3 h pre-incubation to determine the initial population size and after the following 3 h co-incubation period to assess the viability of both organisms (Fig. 1). Ten μL taken from four wells that constitute one sample were pooled and serially diluted in 0.9% NaCl. 100 μL aliquots of 10^− 5^, 10^− 6^ and 10^− 7^ dilutions were plated on LB agar plates supplemented with 12 μg/mL aztreonam (Merck, Switzerland) to eliminate *P. aeruginosa*, and on LB agar plates supplemented with 10 μg/mL vancomycin (Vancocin™, Sandoz Pharmaceuticals, Switzerland) to eliminate *S. aureus*. Colony-forming units (CFU) were counted after 18 h of incubation at 37 °C.

### RNA extraction

The RNeasy Mini Kit (Qiagen, Switzerland) was used for RNA isolation according to the manufacturer’s protocol, which was modified to include combined lysozyme and lysostaphin (Sigma-Aldrich, USA) treatments. Once the short-term competition assay was completed, all samples were centrifuged at 6000 RPM for 5 min at 4 °C and the supernatant was discarded. Pellets were rinsed with 250 μL of an acetone-ethanol (1:1) solution. Tubes containing the cell pellets were stored at − 80 °C. The next day, samples were thawed on ice, centrifuged at 6000 RPM for 10 min at 4 °C. Remaining liquid was removed carefully by pipetting. The pellet was resuspended in 1 mL of TE (10 mM Tris, 1 mM EDTA) buffer at pH 8.0. All samples were again centrifuged at 6000 RPM for 10 min at 4 °C and the supernatant discarded. The pellet was resuspended in 100 μL of lysozyme solution prepared by dissolving 3 mg of lysozyme (Sigma-Aldrich, USA) in 1 ml of Tris-EDTA pH 8.0. Lysostaphin was added to the solution at a final concentration of 1 mg/mL and the samples were incubated at 37 °C for 5 min. From this step onward the manufacturer’s protocol (RNeasy Mini Kit RNA) was followed except that the final elution step was repeated once to increase RNA yield. DNase was removed by digestion with RQ1 RNase-Free DNase (Promega, USA) according to the manufacturer’s protocol. Another RNA cleanup was performed after the DNase treatment. The RNA concentration was then measured using a Nanodrop 1000 spectrophotometer (ThermoScientific, USA).

### RNA sequencing and bioinformatics analysis

A total RNA amount of 1.3 μg was ribodepleted for each replicate sample using the Ribo-Zero rRNA removal Kit for bacteria (Epicentre, USA) following the manufacturer’s protocol. The TruSeq total RNA stranded kit (Illumina, USA) was used to prepare the libraries. The quantity of libraries was determined in a Qubit spectrophotometer and their quality assessed with a Tapestation on a DNA High sensitivity chip (Agilent Technologies, USA). The 18 libraries generated were pooled at equimolarity and loaded at 7 pM for clustering. The samples were sequenced in single reads of 100 bp using TruSeq SBS HS v3 chemistry on an Illumina HiSeq 2500 sequence (Illumina, USA). The sequencing quality control was done with FastQC (http://www.bioinformatics.babraham.ac.uk/projects/fastqc), and all samples passed. Reads were mapped with the TopHat v.2 software [56] to the *P. aeruginosa* UCBP-PA14 RefSeq genome NC_008463.1 for samples PM1, PM2, PM3, and to the *S. aureus* Newman RefSeq genome NC_009641.1 for samples SM1, SM2, SM3, and a merge of the two for samples CC1, CC2, CC3. The tables of counts with the number of reads mapping to each gene feature were prepared with HTSeq v0.5.3 (https://htseq.readthedocs.io/en/release_0.11.1). The counts were normalized according to the library size and genes with less than one count per million reads (cpm) in all three samples were removed. The raw gene number of the set is 5977 for *P. aeruginosa (*analysis CC versus PM), and 2589 for *S. aureus* (analysis CC versus SM). Poorly or unexpressed genes were filtered out. The filtered data set consists of 5403 genes for *P. aeruginosa* PAO1 and 2418 genes for *S. aureus*. Additional file 5: Table S3 shows differentially expressed genes, using a False Discovery Rate of ≤5% (FDR).

### Quantitative real-time PCR

Quantitative Real-Time PCR (qRT-PCR) was performed using the SYBR Green PCR Kit (Qiagen, Switzerland) in a Rotor-Gene 3000 Real-Time PCR machine (Corbette Research, Australia). Specific qRT-PCR primers are listed in Additional File 6: Table S4. The reaction mix was prepared in a final volume of 25 μL: 12.5 μL of Rotor-Gene SYBR Green PCR Master Mix™ 2X, 2.5 μL of forward primer, 2.5 μL of reverse primer, 1 μL of cDNA template diluted to 10 ng/μL in RNase-free water and 6.5 μL of RNase-free water. The program was as follows: 5 min at 95 °C, 5 s at 95 °C, 10 s at 60 °C (40 cycles). Melt curve analyses were performed at the end of the run by heating from 60 to 95 °C. Samples were run in triplicates. Standard curves were prepared by 10-fold dilutions of purified gDNA of *P. aeruginosa* and *S. aureus*. The obtained concentrations for each target gene were then normalized to the concentration of a housekeeping reference gene: *rpsL* for *P. aeruginosa* [57], NWMN_1382 for *S. aureus* [58]. Relative gene expression was calculated for each replicate by dividing the ratio of the target gene over reference gene in co-culture by the same ratio in mono-culture. Relative gene expression is expressed in base 2-logarithms.

## Additional files

Additional file 1: **Figure S1.** MA plots of the differentially expressed genes. (PDF 1068 kb)

Additional file 2: **Figure S2.** Validation of RNAseq data. (PDF 269 kb)

Additional file 3: **Table S1.** Differentially expressed genes in *S. aureus* in co-culture versus mono-culture (average of three replicates, after normalization). (XLSX 35 kb)

Additional file 4: **Table S2.** Differentially expressed genes in *P. aeruginosa* in co-culture versus mono-culture (average of three replicates, after normalization). (XLSX 36 kb)

Additional file 5: **Table S3.** RNAseq results and mapping statistics. (DOCX 15 kb)

Additional file 6: **Table S4.** Primers used in this study. (DOCX 18 kb)

## Abbreviations

CC: Co-culture
CF: Cystic fibrosis
FDR: False discovery rate
HQNO: 2-heptyl-4-quinolone *N*-oxide
NO: Nitric oxide
PM: *Pseudomonas aeruginosa* mono-culture
PQS: Pseudomonas quinolone signal (2-heptyl-3-hydroxy-4-quinolone)
qRT-PCR: Quantitiative RealTime-polymerase chain reaction
QS: Quorum sensing
RNS: Reactive nitrogen species
ROS: Reactive oxygen species
SM: *S. aureus* mono-culture
