# Supplementary material for: Transcriptional profiling of Pseudomonas aeruginosa and Staphylococcus aureus during in vitro co-culture
Source: BMC Genomics. 2019 Jan 10;20:30. doi: 10.1186/s12864-018-5398-y (PMC6327441; doi:10.1186/s12864-018-5398-y)
Supplement: Supplementary file 1 — Figure S1. MA plots of the differentially expressed genes. (PDF 1068 kb) [file 12864_2018_5398_MOESM1_ESM.pdf]

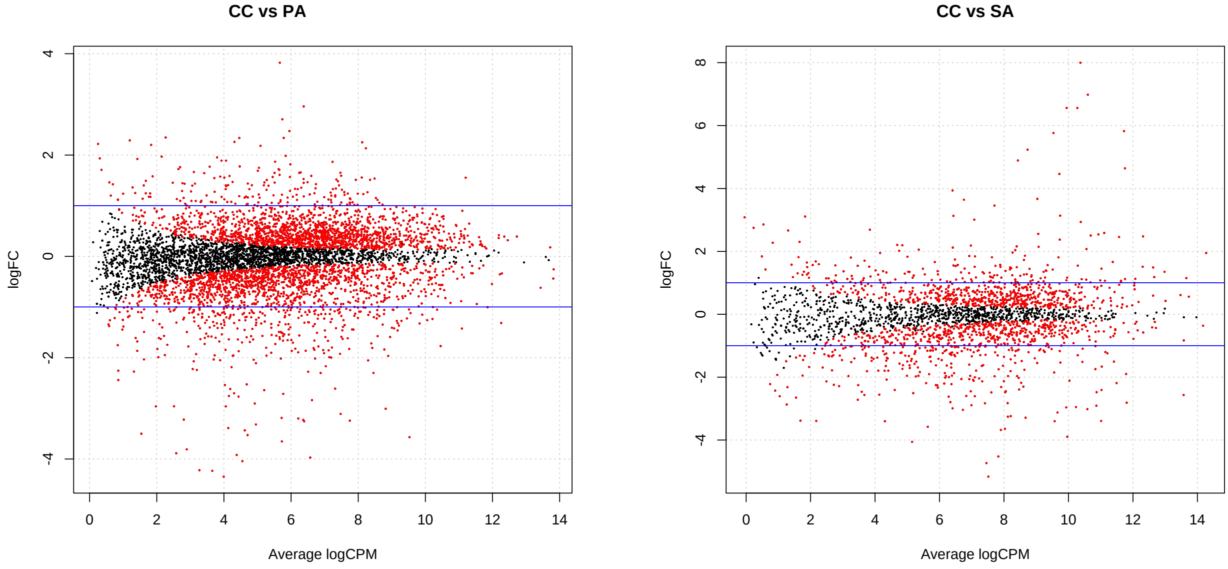


Fig. S1 MA plots of the differentially expressed genes. Differentially expressed genes with a p-value inferior to 0.05 are shown in red. The blue line indicates the FC 2 and -2 (log2 FC >1 and <-1) threshold. The logCPM (count per million) is the average log2 count per million for a given gene taken over all the libraries in the dataset. Co-culture (CC) versus *P. aeruginosa* (PA) mono-culture (left panel) and co-culture (CC) versus *S. aureus* (SA) mono-culture (right panel).
