# Supplementary material for: Transcriptional profiling of Pseudomonas aeruginosa and Staphylococcus aureus during in vitro co-culture
Source: BMC Genomics. 2019 Jan 10;20:30. doi: 10.1186/s12864-018-5398-y (PMC6327441; doi:10.1186/s12864-018-5398-y)
Supplement: Supplementary file 2 — Figure S2. Validation of RNAseq data. (PDF 269 kb) [file 12864_2018_5398_MOESM2_ESM.pdf]

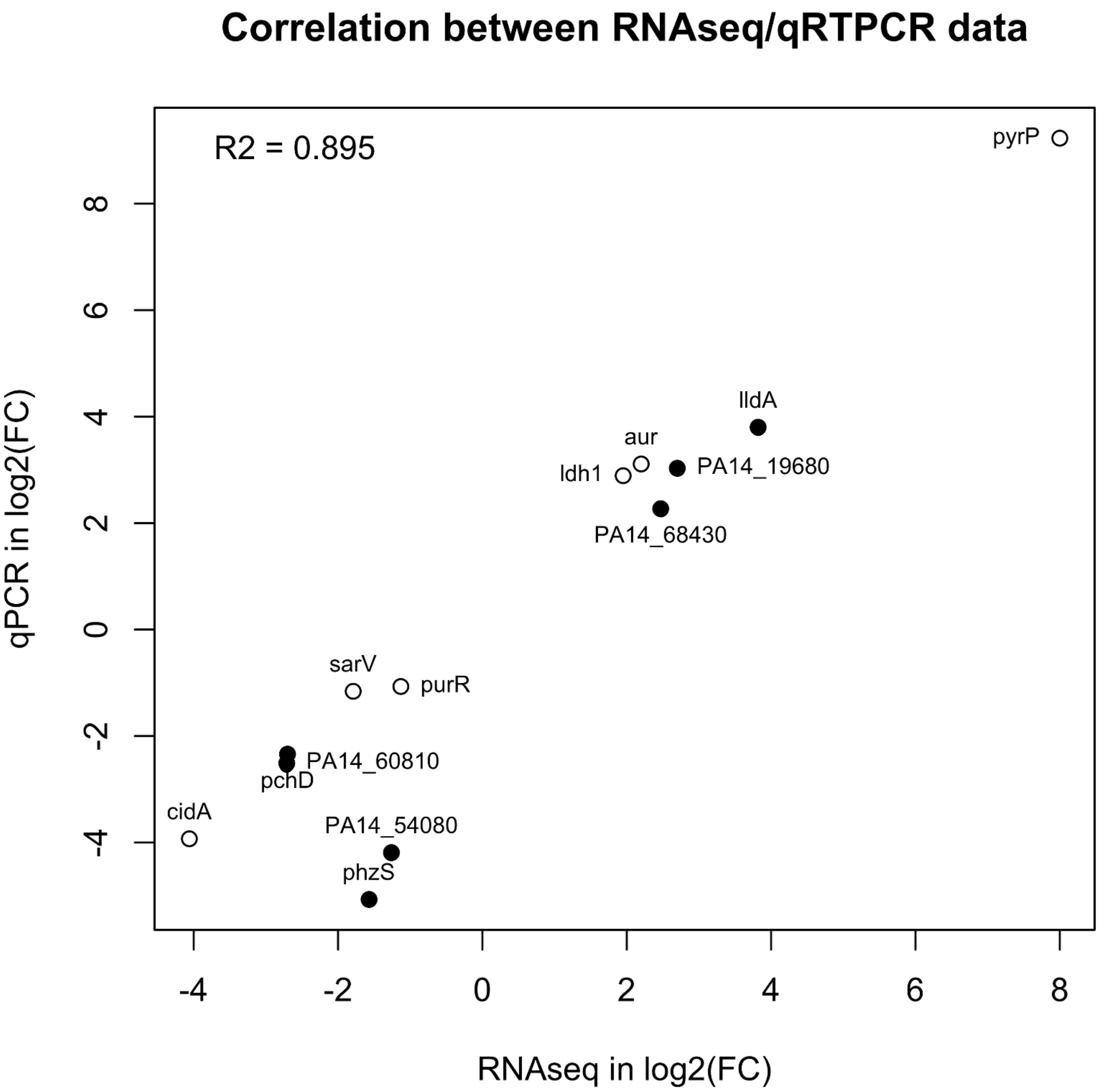

Fig. S2 Comparison of the fold-changes (log_2 base) obtained by RNA sequencing and qRT-PCR for three genes that were up- or down-regulated in each organism in response to the competitor. A statistically significant correlation ($R^2 = 0.895$, $p < 0.05$) was found for the 12 genes tested. *P. aeruginosa* genes (filled symbols), *S. aureus* genes (open symbols). Data points represent the average of the three replicate samples. The package 'gplots' of R (https://cran.r-project.org/web/packages/gplots/index.html) was used for the regression analysis.
