# Supplementary material for: Transcriptional profiling of Pseudomonas aeruginosa and Staphylococcus aureus during in vitro co-culture
Source: BMC Genomics. 2019 Jan 10;20:30. doi: 10.1186/s12864-018-5398-y (PMC6327441; doi:10.1186/s12864-018-5398-y)
Supplement: Supplementary file 5 — Table S3. RNAseq results and mapping statistics. (DOCX 15 kb) [file 12864_2018_5398_MOESM5_ESM.docx]

**Table S3**. RNAseq results and mapping statistics

|  | **SM_1** | **SM_2** | **SM_3** | **PM_1** | **PM_2** | **PM_3** | **CC_1** | **CC_2** | **CC_3** |
| --- | --- | --- | --- | --- | --- | --- | --- | --- | --- |
| **Reads number** | 10’400’115 | 10’807’609 | 14’256’197 | 11’322’303 | 11’598’941 | 12’171’332 | 12’667’559 | 9’979’166 | 11’588’758 |
| **TopHat aligned reads number** | 9’683’935 | 10’089’000 | 13’196’804 | 10’098’725 | 10’258’643 | 10’739’058 | 11’194’873 | 8’848’144 | 10’398’897 |
| **Reads with multiple alignments (%)** | 0.5 | 0.6 | 0.6 | 1.2 | 1.1 | 1.3 | 1.7 | 1.7 | 1.5 |
| **Overall read alignment rates (%)** | 93.1 | 93.4 | 92.6 | 89.2 | 88.4 | 88.2 | 88.4 | 88.7 | 89.7 |

SM, *S. aureus* Mono-culture; PM, *P. aeruginosa* Mono-culture; CC, Co-Culture
