# Supplementary material for: Transcriptional profiling of Pseudomonas aeruginosa and Staphylococcus aureus during in vitro co-culture
Source: BMC Genomics. 2019 Jan 10;20:30. doi: 10.1186/s12864-018-5398-y (PMC6327441; doi:10.1186/s12864-018-5398-y)
Supplement: Supplementary file 6 — Table S4. Primers used in this study. (DOCX 18 kb) [file 12864_2018_5398_MOESM6_ESM.docx]

| **Bacterial species** | **Gene** | **Primer** | **5’-3’ Sequence** | **Reference** |
| --- | --- | --- | --- | --- |
| *S. aureus* | *aur* | aur_F_377 | TTGCAACCGAGTGTTGATGG | This study |
|  |  | aur_R_378 | TGTCGGCTGCGTCATCTTTA | This study |
|  | *pyrP* | pyrP_F_379 | CGATGTTTGGCGCAACAGTA | This study |
|  |  | pyrP_R_380 | CCCGTGATAATTGGCGTGAT | This study |
|  | *ldh1* | ldh1_F_381 | AAGCGAAGCGTTCGATGTTG | This study |
|  |  | ldh1_R_382 | TTGTTCAATTTGCGCTTTGC | This study |
|  | *cidA* | cidA_F_383 | ATCTTCCCTTAGCCGGCAGT | This study |
|  |  | cidA_R_384 | GCGTAATTTCGGAAGCAACA | This study |
|  | *purR* | purR_F_385 | TGGCAAATGCGGTTGCTAAT | This study |
|  |  | purR_R_386 | GCCACCAGCCCTCATAAAATC | This study |
|  | *sarV* | sarV_F_387 | GCGGTAAAGAATTGAGGGATA | This study |
|  |  | sarV_R_388 | TCATCCGTTTCAGAACGCAAT | This study |
|  | NWMN_1382 | HU_1687_F | GGTTTCGGTAACTTTGAGG | Khemici *et al.* 2015 |
|  |  | HU_1747_R | CAGTTTGAGGGTTACGACC | Khemici *et al.* 2015 |
| *P. aeruginosa* | *lldA* | lldA_F_389 | CTGTTCCCTGGAGGACATCG | This study |
|  |  | lldA_R_390 | ATCTGCAGGTCGAGGGTCAG | This study |
|  | PA14_19680 | 19680_F_391 | CGGTACTCATCGCCTTCTGG | This study |
|  |  | 19680_R_392 | CCGGGATGAAGAACAGCAAC | This study |
|  | PA14_68430 | 68430_F_393 | CTGAACCAGGCGGTGATGAT | This study |
|  |  | 68430_R_394 | GCGCTTGAGGTTCCAGAATG | This study |
|  | PA14_60810 | 60810_F_395 | GAGTACCCTCGAACGCCTCA | This study |
|  |  | 60810_R_396 | TCCAGGGCTTCGACATAGGA | This study |
|  | *phzS* | phzS_F_397 | CTGCGCGAATACGAAGAAGC | This study |
|  |  | phzS_R_398 | GTTCCACCTGGTTGCGGTAG | This study |
|  | PA14_54080 | 54080_F_399 | AGCCCTTCGACATCAACGAA | This study |
|  |  | 54080_R_400 | CAGCGGAGGATGAAATCGAG | This study |
|  | *pchD* | pchD_F_412 | AGTTGCAACAGGTGCTGGGAATG | This study |
|  |  | pchD_R_413 | TAGTAGCCGCGGATGGTGTAGGG | This study |
|  | *rpsL* | rpsL_F_308 | GCAAGCGCATGGTCGACAAGA | Dumas *et al.* 2006 |
|  |  | rpsL_R_308 | CGCTGTGCTCTTGCAGGTTGTGA | Dumas *et al.* 2006 |

**Table S4.** Primers used in this study
